# Sequence-Encoded Aggregation of AA10 LPMO Domains as a Basis for Inclusion Body Design

**DOI:** 10.3390/ijms27031188

**Published:** 2026-01-24

**Authors:** Ahmad Muaaz Hassan Butt, Anwar Sunna

**Affiliations:** 1 School of Natural Sciences, Macquarie University, Sydney, NSW 2109, Australia; ahmad.butt@hdr.mq.edu.au; 2 Australian Research Council Industrial Transformation Training Centre for Facilitated Advancement of Australia’s Bioactives (FAAB), Sydney, NSW 2109, Australia

**Keywords:** inclusion bodies, protein self-assembly, aggregation-prone motifs, histidine brace, biofunctional nanomaterials, AA10 homologues

## Abstract

Inclusion bodies (IBs) in *Escherichia coli* are increasingly recognised as nanostructured materials with tunable morphology and functional potential. The N-terminal auxiliary activity family 10 (AA10) lytic polysaccharide monooxygenase (LPMO) domain from *Caldibacillus cellulovorans* (Ccel_p40_) consistently forms IBs and, when fused to diverse proteins, generates functional IBs. Here, we examined whether this strong aggregation propensity is unique to Ccel_p40_ or a broader feature of AA10 LPMOs. Four homologous domains from phylogenetically distinct microorganisms, *Kallotenue papyrolyticum* (Kpap_p40_), *Kibdelosporangium aridum* (Kari_p40_), *Archangium lipolyticum* (Alip_p40_), and *Phytohabitans suffuscus* (Psuf_p40_), were heterologously expressed in *E. coli* under identical cytosolic conditions. All homologues accumulated predominantly in the insoluble fraction, forming morphologically uniform IBs with sub-micron diameters (550–860 nm) and moderate polydispersity indices (0.45–0.54). SDS-PAGE densitometry indicated that most of each expressed protein partitioned into the insoluble fraction. Field-emission scanning electron microscopy revealed compact spherical aggregates, and Fourier-transform infrared spectroscopy showed β-sheet-enriched secondary structures characteristic of ordered IBs. These results indicate that the pronounced aggregation tendency previously observed for Ccel_p40_ is conserved across the AA10 homologues examined. The findings support the view that the AA10 domain represents a promising scaffold for generating stable, recyclable protein nanoparticles and provides a comparative basis for future IB-based biotechnological designs.

## 1. Introduction

Inclusion bodies (IBs) formed during recombinant protein production in *Escherichia coli* were historically viewed as aggregates of misfolded, inactive protein. However, over the past two decades, their perception has evolved substantially. IBs are now recognised as nanostructured protein assemblies with ordered intermolecular architecture and, in many cases, retained biological function [1,2,3,4,5]. These aggregates are often enriched in β-sheet structures and display amyloid-like features, and can be engineered to act as functional catalysts, immobilised biocatalysts, or delivery materials. Their mechanical robustness, high protein density, and ease of recovery make them attractive as catalytic IBs (CatIBs) for applications ranging from enzyme recycling to nanobiomaterial development [6,7]. Recent studies have further demonstrated the utility of IBs as immobilised enzyme systems for robust continuous-flow biocatalysis [8] and for sustainable process design in flow-based bioprocesses [9]. Their reusability and physical stability position them as promising alternatives to conventional immobilisation matrices, expanding their potential well beyond laboratory systems.

Advances in the past decade have shown that IB formation is not solely a random consequence of overexpression stress, but sequence-encoded aggregation can strongly contribute to reproducible aggregation behaviour [1,2,10]. Aggregation-prone sequence motifs, particularly those promoting intermolecular β-strand interactions, contribute to ordered IB formation and can be predicted computationally using bioinformatic prediction tools such as AGGRESCAN [10]. The ability to rationally modulate or exploit these intrinsic aggregation propensities has opened opportunities to design functional IBs with engineered properties [4,11]. This trend has stimulated renewed interest in exploiting aggregation-prone domains as designable scaffolds for biocatalytic nanomaterials [11].

Parallel to this development, lytic polysaccharide monooxygenases (LPMOs) have emerged as a major enzyme class in oxidative biocatalysis. These copper-dependent monooxygenases cleave glycosidic bonds in recalcitrant polysaccharides such as cellulose and chitin through oxidative mechanisms [12,13,14,15,16,17]. LPMOs are classified within the auxiliary activity (AA) families of the CAZy database, with AA10 comprising predominantly bacterial enzymes. The AA10 active site contains a conserved N-terminal histidine brace coordinating a single copper ion that catalyses substrate oxidation [14,17]. Despite their mechanistic sophistication and industrial relevance, heterologous expression of LPMOs in *E. coli* is often challenging. LPMO folding is strongly influenced by coordinated copper loading and periplasmic targeting, and disruption of these processes has been linked to misfolding, instability, or aggregation in heterologous hosts [17,18,19,20].

Our previous work showed that the N-terminal AA10 domain of *Caldibacillus cellulovorans* (Ccel_p40_), originally described as part of a multidomain β-1,4-mannanase [21], consistently forms IBs when expressed in *E. coli*, even when fused to diverse proteins, enzymes, and bioactive peptides [22]. Remarkably, these fusion constructs yield functional inclusion bodies retaining measurable catalytic or biological activity under non-denaturing conditions. The ability of Ccel_p40_ to drive the formation of ordered and active IBs suggested that its strong aggregation propensity is intrinsic to the domain; however, whether this behaviour extends to other AA10 LPMOs remains untested.

Building on this hypothesis, the present study investigates whether the aggregation behaviour observed for Ccel_p40_ is conserved among other AA10 members. To test this, four phylogenetically distinct homologues, *Kallotenue papyrolyticum* (Kpap_p40_), *Kibdelosporangium aridum* (Kari_p40_), *Archangium lipolyticum* (Alip_p40_), and *Phytohabitans suffuscus* (Psuf_p40_), were expressed in *E. coli*. Comparative analyses of their solubility, morphology, and structural features were performed using SDS-PAGE, dynamic light scattering (DLS), field-emission scanning electron microscopy (FESEM), and Fourier-transform infrared (FTIR) spectroscopy. The results presented here reveal that in this study high-yield, β-sheet-rich IB formation is a shared property among the AA10 LPMO homologues examined, supporting the view that aggregation may represent an intrinsic property of these examined AA10s rather than a host-specific effect, although host physiology likely modulates the magnitude of aggregation.

## 2. Results

### 2.1. Sequence Analysis of AA10 LPMO p40 Homologues

**Figure 1:**
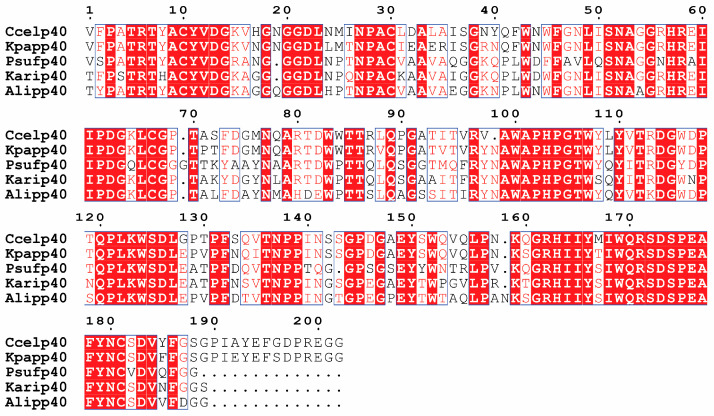
Multiple sequence alignment of the expressed AA10 LPMO catalytic domains of p40 homologs relative to *C. cellulovorans* (Ccel_p40_). The alignment corresponds to the sequences cloned into the expression plasmid and heterologously expressed in *E. coli*. The alignment reflects the expressed catalytic domains following removal of native signal peptides and construct-specific N-terminal residues, including the initiator methionine. Rectangles indicate the aligned AA10 catalytic core region included in each expressed p40 construct. Fully conserved residues are shown as white lettering on a red background, residues conserved by similarity are shown in red, and non-conserved residues are shown in black. The alignment highlights conserved motifs within the AA10 catalytic core, including the conserved internal histidine at approximately alignment position 163, which forms part of the canonical histidine-brace copper-binding site in mature AA10 LPMOs, as well as conserved residues associated with the substrate-binding surface.

Multiple sequence alignment of the expressed AA10 LPMO p40 catalytic domains from *C. cellulovorans* (Ccel_p40_) with four phylogenetically diverse homologs, *Kallotenue papyrolyticum* (Kpap_p40_), *Kibdelosporangium aridum* (Kari_p40_), *Archangium lipolyticum* (Alip_p40_), and *Phytohabitans suffuscus* (Psuf_p40_), revealed strong conservation of motifs within the AA10 catalytic core that are associated with copper coordination and LPMO catalytic activity (Figure 1).

All homologues retained the conserved internal histidine that forms part of the canonical histidine-brace copper-binding site in mature AA10 LPMOs, together with other conserved residues characteristic of the AA10 family. Overall, pairwise identities ranged from 73% (Ccel_p40_–Alip_p40_) to 86% (Ccel_p40_–Kpap_p40_). Sequence differences were primarily confined to surface-exposed loop regions and the C-terminal segments, consistent with the structural variability reported among AA10 family members and with the experimentally observed differences in aggregation behaviour. The full amino acid sequences of the expressed AA10 LPMO p40 homolog constructs are provided in Table S1.

### 2.2. Expression and IB Formation

**Figure 2:**
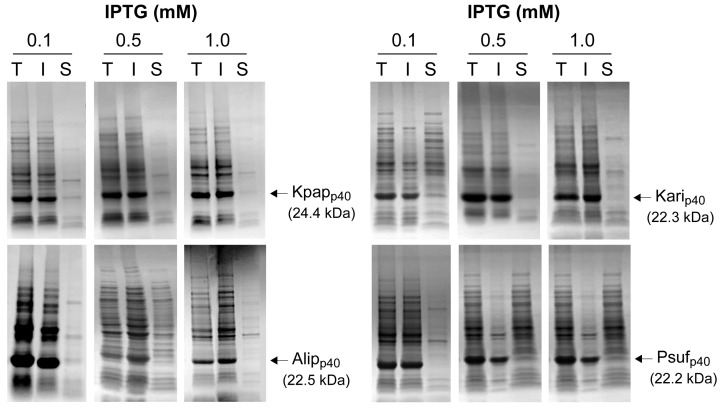
SDS–PAGE analysis of recombinant AA10 LPMO p40 homologue expression and cellular partitioning in *E. coli*. For each homolog, samples correspond to protein fractionation into total (T), insoluble (I), and soluble (S) fractions. Densitometric quantification of p40 band intensities in the insoluble and soluble fractions is summarised in Table 1.

Recombinant expression of the four AA10 LPMO homologs in *E. coli* BL21(DE3) resulted in predominant accumulation of the target proteins in the insoluble fraction, with observed bands corresponding to the expected molecular masses of 22–24 kDa (Figure 2).

Densitometric analysis of SDS–PAGE gels (Figure 2, Table 1) was performed by quantifying the band intensities corresponding specifically to the recombinant p40 homologs in the insoluble (I) and soluble (S) fractions. At 0.1 mM IPTG, the majority of the detected p40 signal was present in the insoluble fraction for all homologs, representing approximately 91–95% of the total p40 band intensity (Table 1). Across the IPTG titration range tested (0.1–1 mM), the p40 signal remained predominantly associated with the insoluble fraction for all homologs. Although modest variations in the relative proportion of soluble protein were observed between IPTG concentrations, no strong or systematic IPTG concentration–dependent trend was evident. These differences are therefore interpreted as minor experimental variability rather than a concentration-driven effect, with robust IB formation observed under all induction conditions tested. Consistent with this behaviour, the very weak bands observed in the soluble (S) fractions reflect the strong aggregation propensity of the AA10 p40 domains under cytoplasmic expression conditions, resulting in preferential partitioning of the recombinant protein into the insoluble fraction.

### 2.3. Partial Purification and Stability of IBs

Sequential washing with 1× PBS (pH 7.5), 0.1% Triton X-100, and the commercial bacterial protein extraction reagent, B-PER, efficiently removed loosely associated impurities while retaining most of the IB material (Figure S1). Retention yields and pellet characteristics after purification are summarised in Table 2. Kpap_p40_ and Karip_40_ IBs remained hard and cohesive throughout the washing sequence, with retention of ~90% or higher at 0.1–0.5 mM IPTG. Alip_p40_ and Psuf_p40_ displayed partial softening at higher induction levels (1 mM IPTG), retaining ~15–45% of their initial mass, indicative of reduced mechanical stability at higher induction levels. The SDS–PAGE analysis confirmed that all IBs retained their dominant 22–24 kDa bands with minimal degradation, supporting the robustness of the purification protocol.

### 2.4. Hydrodynamic Size and Dispersity of IBs

Dynamic light scattering (DLS) analysis revealed submicron-scale IB aggregates with hydrodynamic diameters between 550 and 863 nm and moderate polydispersity (PDI 0.45–0.54) across all variants (Table 3). Mean hydrodynamic diameters and corresponding PDI values for each homologue are summarised in Table 3. All variants displayed broad size distributions, consistent with heterogeneous populations of recombinant IBs in suspension.

### 2.5. Morphology of IBs by FESEM

**Figure 3:**
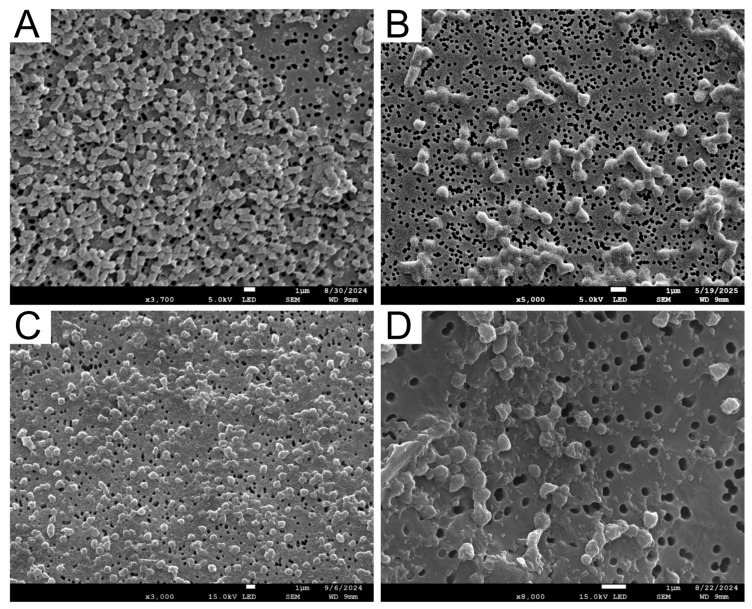
Field-emission scanning electron microscopy (FESEM) images of IBs formed by recombinant AA10 LPMO p40 homologs expressed in *E. coli*. Representative micrographs are shown for (**A**) Kpap_p40_, (**B**) Karip_p40_, (**C**) Alip_p40_, and (**D**) Psuf_p40_. The scale bar corresponds to 1 µm in all images. Quantitative FESEM measurements are reported in Table 4.

FESEM analysis showed that all AA10 LPMO IBs formed compact, spherical to ellipsoidal particles (Figure 3). Kpap_p40_ and Kari_p40_ IBs appeared relatively uniformly distributed and densely packed, with particle dimensions predominantly in the submicron range, broadly comparable to the hydrodynamic sizes measured by DLS. In contrast, Alip_p40_ and Psuf_p40_ exhibited broader particle-size distributions with irregular surface features, frequently forming loosely associated clusters. As FESEM reports particle Feret diameters of dried samples, while DLS measures hydrodynamic diameters of hydrated aggregates, exact correspondence between the two techniques is not expected. Nevertheless, both methods consistently indicate submicron-scale IBs across all variants.

Quantitative image-based measurements (Table 4) further indicate that Kpap_p40_ and Kari_p40_ IBs tend to form slightly more elongated particles, whereas Alip_p40_ and Psuf_p40_ display greater size variability.

### 2.6. In Silico Prediction of Aggregation Propensity

To further assess whether the observed aggregation behaviour is encoded in the primary sequence, sequence-based aggregation-propensity analysis was performed for all AA10 LPMO p40 homologs using the AGGRESCAN web server. AGGRESCAN identifies contiguous amino-acid clusters with high intrinsic β-aggregation potential and reports quantitative indices including total hotspot area (THSA), number of hotspots (nHS), and the normalised aggregation score (Na_4_vSS) (Table S2). All homologs displayed multiple aggregation-prone regions, including conserved hotspots immediately downstream of the N-terminal histidine-brace motif and within β-strand segments of the catalytic core. While the positions of several hotspots were conserved, the overall aggregation profiles differed quantitatively between homologs. In particular, Alip_p40_ and Psuf_p40_ exhibited the highest THSA and Na_4_vSS values, whereas Kpap_p40_ and Kari_p40_ showed lower aggregate scores.

This ranking (Psuf_p40_ ≈ Alip_p40_ > Kpap_p40_ ≈ Kari_p40_) is consistent with experimental observations from DLS and FESEM, where Psuf_p40_ and Alip_p40_ formed larger and more polydisperse IBs, while Kpap_p40_ and Kari_p40_ produced smaller, more compact particles. These results indicate that aggregation propensity is at least partly intrinsic to the AA10 LPMO catalytic domain sequence, rather than arising solely from heterologous overexpression conditions in *E. coli*. Exact residue positions and properties of predicted aggregation hotspots are provided in Table S2 to enable direct comparison across homologues.

### 2.7. Secondary-Structure Characteristics by FTIR

FTIR spectra of all IB preparations showed three characteristic bands corresponding to Amide I (1650 cm^−1^), Amide II (1540 cm^−1^), and Amide III (1240 cm^−1^) (Figure S3). The Amide I band mainly originates from C=O stretching vibrations, whereas the Amide II and Amide III bands are associated with N–H bending and C–N stretching motions of the peptide backbone [23]. The Amide I envelope centred at 1650 cm^−1^ reflects the overall backbone conformation, while deconvolution revealed a β-sheet-associated component in the 1620–1630 cm^−1^ region. Quantitative deconvolution (Table 5) showed that all IBs were β-sheet-rich (41–52%), consistent with ordered aggregation while retaining native-like secondary-structure elements. Slight variations in Amide I peak sharpness and intensity were observed among homologs: Kpap_p40_ and Kari_p40_ exhibited narrower, more intense β-sheet peaks, whereas Alip_p40_ and Psuf_p40_ showed broader profiles, suggesting greater structural heterogeneity. These differences align with the DLS and FESEM results, indicating that all four AA10 LPMOs form structurally ordered yet distinct IBs assemblies.

## 3. Discussion

The present study demonstrates that aggregation into IBs is a recurring feature among the AA10-family homologs examined here when heterologously expressed in *E. coli*. Although only five AA10 catalytic domains have been analysed to date, the consistent observation of predominant insolubility across phylogenetically diverse homologues suggests that strong aggregation propensity may not be unique to Ccel_p40_, but may instead reflect a more widespread, sequence-encoded characteristic of AA10 catalytic domains when expressed outside their native secretion and metal-loading context. The four homologs Kpap_p40_, Kari_p40_, Alip_p40_, and Psuf_p40_ all accumulated predominantly as IBs, with yields exceeding 85% of the total expressed protein, showing little dependence on induction strength. These findings extend earlier observations on *C. cellulovorans* Ccel_p40_, previously shown to possess an intrinsic aggregating property and a tendency to form IBs in *E. coli* [22]. They further support the view that insoluble aggregate formation in this system is not simply an artefact of host stress or overexpression but may reflect sequence-encoded features of these AA10 catalytic domains.

Consistent with this view, several bacterial AA10 LPMOs are difficult to obtain as soluble, correctly folded proteins in *E. coli* and are often produced as IBs, with solubility frequently improved only through the use of appropriate secretion signals or folding aids [18,19,24]. A recent review by Zhang et al. [24] summarised heterologous expression of AA10s from diverse bacterial genera, including *Bacillus*, *Enterococcus*, *Cellulomonas*, *Cellvibrio*, *Chitinolyticbacter*, *Hahella*, *Streptomyces*, *Thermobifida*, and *Kitasatospora*, and highlighted common challenges such as incorrect folding, IB formation, misselection of signal peptides, and low extracellular yields in *E. coli*. Qin et al. [25] further showed that the *Streptomyces megaspores* AA10 SmLpmo10A initially accumulated as IBs in *E. coli* Transetta (DE3) and required co-expression of molecular chaperones in Origami (DE3) and Shuffle T7-B strains to achieve soluble expression, directly illustrating the need for chaperone-assisted folding for some AA10s. In line with this, Gaber et al. [18] reported that the AA10 TtAA10A from the shipworm symbiont *Teredinibacter turnerae* could only be produced efficiently in *E. coli* after co-expression of the pGro7 chaperone plasmid, following multiple unsuccessful attempts using different solubility tags and secretion signals. Together, these studies show that AA10 LPMOs from a range of bacterial genera may require, or substantially benefit from, secretion pathways and/or chaperone systems to obtain properly folded, functional enzymes.

Sequence analysis showed that all homologs share hallmark AA10 motifs within the catalytic core, including conserved residues associated with copper coordination and oxygen activation in mature AA10 LPMOs [12,13]. Structural studies have established that this region, together with adjacent β-strand loops surrounding the metal site, forms an exposed hydrophobic platform essential for polysaccharide depolymerisation [12,15]. These loops, particularly the flexible L2 and neighbouring surface segments, constitute a structurally diverse region that forms a major part of the substrate-binding surface [26] and, in cellulose-active AA10s, frequently contain hydrophobic residues that contribute to substrate engagement [13,14,27]. Although this hydrophobicity and mobility are functionally important, any contribution to self-association under incomplete folding or metal-loading conditions remains a plausible interpretation rather than a demonstrated mechanism.

Our in silico aggregation hotspot analysis is consistent with this interpretation. Aggregation-prone sequences were consistently located immediately downstream of the region corresponding to the histidine-brace motif in mature AA10 LPMOs, and within β-sheet regions bordering the catalytic pocket. These predicted hotspot regions overlap with the L2 and neighbouring β-strand-associated segments identified as variable and surface-exposed in AA10s crystal structures [14]. The recurrence of aggregation-prone motifs in corresponding regions across multiple homologues supports the hypothesis that aggregation propensity may represent an intrinsic feature of the AA10 catalytic fold itself, rather than an isolated anomaly of individual sequences. This correspondence supports the view that, in these AA10s, residues involved in catalysis occupy spatial regions that also exhibit features commonly associated with aggregation propensity. This aligns with structural reviews highlighting the close relationship between catalytic function and surface hydrophobicity of AA10s [12,13,27].

A notable contrast arises when comparing the present cytoplasmic expression results with those of Forsberg et al. [19], who examined the same N-terminal AA10 catalytic domain from the multi-modular *C. cellulovorans* ManA enzyme originally described by Sunna et al. [21]. In their study, soluble and catalytically active enzyme was obtained only when the construct was directed to the periplasm using a signal peptide, where cleavage generated the mature N-terminal histidine essential for copper binding. Periplasmic expression therefore enables correct N-terminal processing [18,19,24,28] and thereby exposes the N-terminal histidine that forms the histidine braces required for copper coordination [13,14,18,24,29], and supports efficient copper loading at this site [18,19]. When expressed without a signal peptide, as in the present work, the domain accumulates in the reducing cytoplasm, where incomplete copper coordination likely contributes to misfolding and aggregation, while any involvement of off-pathway oxidation remains a plausible possibility rather than a demonstrated mechanism. This interpretation is consistent with the folding–redox coupling model, which proposed that copper coordination and oxidative maturation are tightly linked and that redox imbalance can lead to self-oxidation and irreversible inactivation [16,20,30,31,32]. These observations support the view that cytoplasmic aggregation of AA10 domains reflects factors normally stabilised by secretion-associated processing and copper loading, while evidence from heterologous systems indicates that some AA10s may also depend on chaperone-assisted folding [18,24].

The physicochemical properties of the resulting IBs further substantiate their ordered nature. DLS revealed sub-micron aggregates ranging from 550 to 863 nm with moderate polydispersity (PDI 0.45–0.54), values that fall within the nanoscale size range reported for bacterial IBs and functional IB-derived nanoparticles [33,34,35,36]. Polydispersity differed among the homologues, following the trend Alip_p40_ > Psuf_p40_ > Kari_p40_ ≈ Kpap_p40_, indicating homolog-dependent differences in particle heterogeneity. These observations support their classification as ordered nanostructures rather than amorphous precipitates [4,6,37,38]. FESEM showed densely packed spherical to ellipsoidal particles with homolog-specific variation in compactness, which we interpret as reflecting sequence-dependent differences in physicochemical properties. The combined FESEM and FTIR signatures agree with those reported for ordered amyloid-like IBs [4,6,7,36,39,40,41]. FTIR spectra displayed prominent Amide I envelope centred around 1650 cm^−1^, and deconvolution revealed a β-sheet-associated component in the 1620–1630 cm^−1^ region, consistent with our calculated β-sheet contents of 41–52%. This aligns with the β-sheet-rich and partially folded conformations characteristic of functional IBs [4,6,7,36,39,40,41].

Homolog-specific differences in particle morphology correlate with predicted aggregation propensities. AGGRESCAN analysis identified a greater number and cumulative extent of aggregation hotspots in Alip_p40_ and Psuf_p40_, whereas Kpap_p40_ and Kari_p40_ contained fewer predicted aggregation hotspots. This trend is consistent with experimental observations showing greater heterogeneity and less compact IB morphologies for Alip_p40_ and Psuf_p40_, compared with the more compact IBs formed by Kpap_p40_ and Kari_p40_. This correspondence between sequence-encoded aggregation potential and experimental morphology supports the view that IB formation reflects, at least in part, intrinsic biophysical properties of these domains. Similar protein-dependent relationships between IB morphology and sequence or folding behaviour have been reported for other recombinant proteins, including catalytically active and GFP-based IBs [4,7,34,36,41], reinforcing that the observed aggregation behaviour is primarily sequence- and folding-dependent rather than solely an artefact of overexpression or host stress.

From a mechanistic perspective, AA10 LPMOs possess an immunoglobulin-like β-sandwich core with surface-exposed loops surrounding the copper centre [14,28,42,43]. The β-sandwich core forms a structurally rigid scaffold, while the substrate-binding loops on the catalytic surface exhibit greater intrinsic mobility [29,43] and are central to catalytic function and stability [42]. Although no studies have directly examined how these architectural features affect folding during heterologous expression, the conserved β-sandwich topology is known to impose characteristic folding constraints in Ig-like domains [44], providing a plausible structural basis for differences in folding behaviour when the enzyme is expressed outside its native secretion pathway and metal-loading environment. In native hosts, AA10s are secreted and frequently occur as modular enzymes with carbohydrate-binding modules (CBMs), which orient the catalytic domain on crystalline polysaccharides and modulate catalytic performance [12,28]. CBMs have not been shown to confer direct structural stabilisation, but modular LPMOs display lower susceptibility to rapid oxidative inactivation compared with isolated catalytic domains [45,46,47], indicating a functional protective role. Cytoplasmic expression of isolated AA10 catalytic domains, as observed for CcelAA10 in Forsberg et al. [19], is associated with insolubility, supporting the view that secretion-associated processing and auxiliary modules contribute to proper folding and copper incorporation. The copper redox cycle of LPMOs can generate reactive oxygen species even in the absence of substrate, leading to oxidative modification and inactivation [14,15,20]. Such off-pathway reactions perturb the catalytic site and cause oxidative damage, but a direct mechanistic link to aggregation has not been demonstrated. Consistent with this, Forsberg et al. [19] showed that non-substrate-bound LPMOs undergo oxidative damage and loss of activity. Although the precise relationship between redox chemistry and aggregation remains unresolved, the combined evidence supports a model in which oxidative perturbation, together with the absence of stabilising native interactions, increases the tendency of isolated catalytic domains to misfold and aggregate under reducing cytoplasmic conditions.

Removal of these auxiliary elements and cytoplasmic expression are consistent with the increased aggregation observed for isolated AA10 catalytic domains [18,19,24]. The copper redox cycle of LPMOs can generate reactive oxygen species even in the absence of substrate, leading to oxidative modification and inactivation [14,15,20]. Such off-pathway reactions can perturb the catalytic domain and may create conditions that favour aggregation. Consistent with this, Forsberg et al. [19] demonstrated that off-pathway redox events cause oxidative damage and loss of activity when LPMOs are not substrate-bound. Although the precise relationship between redox chemistry and aggregation remains unresolved, these observations are compatible with a model in which structural perturbation increases aggregation propensity.

These insights also underscore the critical influence of the signal peptide and expression compartment on solubility and activity. Forsberg et al. [19] showed that the same N-terminal AA10 catalytic domain is recovered as a monomeric, copper-loaded, catalytically active enzyme only when exported to the periplasm. In contrast, our cytoplasmic expression experiments resulted in insoluble material that lacked proper N-terminal processing and copper coordination. Our findings additionally show that aggregation behaviour correlates with sequence features within the catalytic domain, and the alignment of predicted aggregation hotspots with regions surrounding the copper-binding site suggests a spatial overlap between residues involved in catalysis and those contributing to aggregation.

The IBs produced here share several characteristics with engineered CatIB systems that have been intentionally developed for biocatalyst immobilisation [3,7,35]. Fusion of aggregation-prone peptides has been shown to generate IBs that are mechanically stable, catalytically active, and recyclable [3,7]. The AA10-derived IBs exhibit similar properties, including uniform nanoscale morphology, ordered secondary structure, and high yield under standard culture conditions. These features, together with their β-sheet order and morphological uniformity, suggest that AA10 domains could be repurposed as self-assembling scaffolds for constructing catalytic IBs or hybrid protein-based nanocomposites.

While IB formation is often viewed as a limitation in recombinant production, the concept of functional IBs reframes aggregation as a useful self-assembly process [35]. The present results provide comparative evidence that this behaviour extends across multiple AA10 family homologs, identifying structural and sequence determinants that govern aggregation propensity. This knowledge offers opportunities for rational design of LPMO-derived scaffolds and for tuning solubility through signal-peptide engineering, domain fusion, or cofactor supplementation.

Functional IBs have been extensively described for a wide range of recombinant proteins [4,6,7]. We have also demonstrated that Ccel_p40_ fusion constructs can generate functional or catalytically active IBs [22]. As the present study focuses specifically on structural and aggregation behaviour, the detailed functional characterisation of these newly generated AA10-derived IBs will be presented elsewhere. Overall, our data establish that AA10 catalytic domains consistently form IBs in the *E. coli* cytoplasm, indicating that aggregation is an intrinsic, sequence-encoded property shared across these homologues. The observed aggregation correlates with conserved hydrophobic and β-sheet features, homolog-specific particle morphology, and the nanoscale uniformity typical of structured protein assemblies. In combination with prior evidence from catalytic IBs [3,4,7] and soluble LPMOs expressed in the periplasm [19], these results identify AA10 domains as promising natural templates for engineering self-assembling biocatalysts and protein-based nanomaterials.

## 4. Materials and Methods

### 4.1. Chemicals and Reagents

All reagents were of analytical grade and purchased from Sigma-Aldrich (St. Louis, MO, USA) unless otherwise specified.

### 4.2. Gene Selection and Sequence Analysis

Four AA10 LPMO homologues were selected to evaluate whether the strong aggregation behaviour previously observed for *Caldibacillus cellulovorans* Ccel_p40_ is widespread within this enzyme family. The sequences were retrieved from the NCBI GenBank database: *Kallotenue papyrolyticum* (Kpap_p40_), *Kibdelosporangium aridum* (Kari_p40_), *Archangium lipolyticum* (Alip_p40_), and *Phytohabitans suffuscus* (Psuf_p40_). Amino-acid sequences corresponding to the expressed catalytic domains (following removal of native signal peptides and construct-specific N-terminal residues) were aligned with the reference Ccel_p40_ using Clustal Omega (EMBL-EBI) (https://www.ebi.ac.uk/jdispatcher/msa/clustalo, accessed on 15 December 2025) to assess conservation of motifs within the AA10 catalytic core, including residues associated with copper coordination and substrate binding. Aggregation-propensity profiles were generated with AGGRESCAN [10] using default parameters, and the total hotspot area (THSA), number of aggregation hotspots (nHS), and average aggregation-propensity score (Na4vSS) were recorded.

### 4.3. Construction of Expression Plasmids

DNA manipulations and restriction digestions were performed following standard molecular biology protocols [48]. The pETDuet-1-p533 expression vector, harbouring the gene encoding the p40 domain of *C. cellulovorans* ManA preceded by a 3×(GGGGS) flexible linker, was first transformed into *E. coli* DH5α competent cells. Positive transformants were identified by colony PCR (cPCR) and confirmed by agarose gel electrophoresis. The p533 vector was subsequently digested with EcoRI and BamHI, and the linearised fragment was separated by agarose gel electrophoresis, excised, and purified using the Monarch DNA Gel Extraction Kit (New England Biolabs, NEB, Ipswich, MA, USA). To prevent self-ligation, the linearised vector was dephosphorylated with Antarctic Phosphatase (NEB) and further purified using the Monarch PCR & DNA Cleanup Kit (NEB), ensuring complete removal of the original p40 insert.

Genes encoding the AA10-LPMO p40 homologues (Alip_p40_, Kpap_p40_, Psuf_p40_, and Kari_p40_) were synthesised as gBlock Gene Fragments (Integrated DNA Technologies, Singapore). Each insert was digested with EcoRI and BamHI to match the restriction sites of the prepared vector and subsequently purified from agarose gels using the Monarch DNA Gel Extraction Kit. Ligation reactions were performed using the Quick Ligation Kit (NEB), and ligation mixtures were transformed into *E. coli* DH5α competent cells. Positive clones were confirmed by cPCR and agarose gel electrophoresis. Verified plasmids were purified and transformed into *E. coli* BL21(DE3) competent cells for heterologous protein expression. Construct identity was further confirmed by DNA sequencing (Macrogen Inc., Seongnam-si, Republic of Korea). The resulting expression plasmids, pETDuet-1-Alip_p40_, pETDuet-1-Kpap_p40_, pETDuet-1-Psuf_p40_, and pETDuet-1-Kari_p40_, were used for subsequent protein expression experiments.

### 4.4. Protein Expression

Recombinant expression of p40-based IBs was performed in *E. coli* BL21(DE3) using Luria–Bertani (LB) medium supplemented with ampicillin (100 µg mL^−1^). An overnight seed culture (5 mL) of cells harbouring the appropriate expression plasmid was used to inoculate 100 mL of fresh LB medium at a 2% (*v*/*v*) inoculation ratio. Cultures were incubated aerobically at 37 °C and 200 rpm in a shaking incubator until reaching mid-logarithmic growth phase (OD_600_ = 0.6–0.8). Protein expression was induced with isopropyl β-D-1-thiogalactopyranoside (IPTG) at final concentrations of 0.1 mM, 0.5 mM, and 1 mM as part of a single IPTG titration experiment to compare IB formation across standard induction conditions. For this titration, all induction conditions were performed using the same recombinant *E. coli* BL21(DE3) clone to minimise inter-clonal variability. A single colony was used to inoculate a pre-culture, which was subsequently split into parallel expression cultures induced with different IPTG concentrations. Following induction, cultures were incubated for an additional 4 h at 37 °C and 250 rpm. Cells were harvested by centrifugation (10,000× *g*, 15 min, 4 °C) and washed once with 1× phosphate-buffered saline (PBS, pH 7.5). The resulting cell pellets were centrifuged again under the same conditions and stored at −30 °C until further use.

For the isolation of IBs, cells were resuspended in 5 mL lysis buffer (50 mM Tris-HCl, 100 mM NaCl, 1 mM EDTA, 1 mM serine protease inhibitor Pefabloc^®^ SC, and 2% glycerol, pH 7.5). The suspension was homogenised by gentle vortexing and subjected to ultrasonic disruption using a Branson 450 Digital Sonifier (Marshall Scientific, Hampton, NH, USA) for 8 min with a 5 s on/10 s off duty cycle at 35% amplitude. After sonication, a small aliquot of the total protein (TP) fraction was collected for subsequent qualitative analysis. The cell lysate was centrifuged at 10,000× *g* for 15 min at 4 °C to separate soluble and insoluble fractions. The supernatant containing the soluble protein (SP) and the pellet containing the insoluble fraction (P) were collected separately. Both fractions were analysed by SDS–PAGE to assess protein expression and solubility, and densitometric quantification of band intensity was performed using ImageJ software (version 1.54p, NIH, Bethesda, MD, USA). All densitometric analyses were performed with background subtraction. The relative yield (%) of each fraction was calculated according to:

Yield (%) = [*A*_n_/*A*_t_] × 100

where *A*_n_ represents the densitometric area of the band corresponding to either the soluble or insoluble protein fraction, and *A*_t_ represents the densitometric area of the total protein fraction as measured in ImageJ.

### 4.5. Sodium Dodecyl Sulfate–Polyacrylamide Gel Electrophoresis (SDS–PAGE)

Protein samples were mixed with an equal volume of Laemmli sample buffer (2×) and incubated at 95 °C for 5 min to achieve complete denaturation. Denatured samples were briefly centrifuged to remove condensate and then loaded onto precast Mini-PROTEAN^®^ TGX gels (4–20%) (Bio-Rad Laboratories, Hercules, CA, USA). Electrophoresis was performed at a constant voltage of 100 V for 60 min using running buffer composed of 25 mM Tris, 192 mM glycine, and 1% (*w*/*v*) sodium dodecyl sulfate (SDS), adjusted to pH 8.3. After electrophoresis, the gels were stained with Coomassie Brilliant Blue G-250 Bio-Safe Stain (Bio-Rad Laboratories, Hercules, CA, USA) and destained with Milli-Q water until clear band visualisation was achieved. Protein bands were imaged using the G:BOX F3 Gel Documentation System (Syngene, Cambridge, UK). Gel images were acquired under non-saturating conditions, with exposure settings adjusted to ensure that pixel intensities remained within the linear dynamic range of the detector, thereby avoiding saturation or blooming effects that could compromise densitometric quantification. The Precision Plus Protein™ Dual Color Standard (Bio-Rad Laboratories, Hercules, CA, USA) was included in all gels as a molecular weight reference.

### 4.6. Partial Purification of IBs

Following cell lysis, the resulting insoluble pellet was initially washed with 1× PBS (pH 7.5) and centrifuged at 10,000× *g* for 15 min at 4 °C to remove loosely associated contaminants. The pellet was then subjected to a detergent wash using 5 mL of 0.1% (*v*/*v*) Triton X-100 (Thermo Fisher Scientific, Scoresby, Victoria, Australia), followed by centrifugation under the same conditions. To eliminate residual detergent, the pellet was rinsed twice with sterile Milli-Q water. For further purification, approximately 1 g of the washed insoluble fraction was incubated with 4 mL B-PER™ reagent (Thermo Fisher Scientific, Scoresby, Victoria, Australia) supplemented with 5 units of Benzonase^®^ nuclease (Sigma-Aldrich, St. Louis, MO, USA) at room temperature for 15 min with gentle mixing. After incubation, the solubilised protein was separated from the IB-rich pellet by centrifugation at 10,000× *g* for 15 min at 4 °C. The pellet, representing the purified insoluble protein fraction, was subsequently washed twice with Milli-Q water and stored at −30 °C for downstream analyses. Protein samples collected at each washing step, including the final partially purified IB fraction, were resuspended in sterile Milli-Q water or 1× PBS and analysed by SDS–PAGE to evaluate protein size, purity, and relative yield following purification.

### 4.7. Protein Quantification

The molar extinction coefficient and molecular weight of each IB were calculated from the deduced amino acid sequence using the ExPASy ProtParam tool (https://web.expasy.org/protparam/, accessed on 4 February 2025). A total of 3 mg of each IB preparation was resuspended in 300 µL of solubilisation buffer (50 mM Tris-HCl, pH 8.5, 5 mM dithiothreitol). Suspensions were pipetted and vortexed vigorously until no visible clumps remained. Aliquots of 100 µL were transferred into three tubes to enable triplicate measurement. Samples were further solubilised by addition of solubilisation buffer containing 1–3 M guanidinium hydrochloride (GdmCl) to yield a final IB concentration of 1 mg/mL. After incubation at room temperature for 2 h with gentle agitation, samples were centrifuged (12,000× *g*, 20 min, 4 °C) to remove residual insoluble material. The absorbance at 280 nm (A_280_) was measured using a NanoDrop One/One^c^ spectrophotometer (Thermo Fisher Scientific) after blanking against the solubilisation buffer containing the same GdmCl concentration, with baseline correction at 350 nm. Protein concentration was calculated using the Beer–Lambert law:

C_protein_ (M) = A_280_/ε_protein280_ × l

where C_protein_ is the molar concentration of protein, A_280_ is the absorbance at 280 nm, ε_protein280_ is the theoretical molar extinction coefficient (calculated using ProtParam), and l is the optical pathlength (cm).

The protein content (%) of each IB preparation was calculated as:

Protein content_IBs_ [%] = [Protein concentration_IBs_ (mg mL^−1^)/Amount of IB pellet (mg mL^−1^)] × 100

All measurements were performed in triplicate, and results are reported as mean ± SD values.

### 4.8. Dynamic Light Scattering (DLS)

The hydrodynamic diameter of IBs was determined by dynamic light scattering (DLS) using a Zetasizer Nano ZSP (Malvern Instruments Ltd., Worcestershire, UK) equipped with a 50 µL quartz cuvette. IB suspensions were diluted 50–100 fold with sterile Milli-Q water or 1× PBS (pH 7.0) prior to measurement. Analyses were performed at 25 °C using disposable cuvettes, after 1 min equilibration, with the refractive index of wate r (dispersant) set to 1.33 and material to 1.45. Measurements were collected in normal resolution mode, and particle size distributions were expressed as the diameter of an equivalent hydrodynamic sphere. Each sample was measured in triplicate, and the reported hydrodynamic diameter represents the mean ± standard deviation. Water and 1× PBS were used as dispersants in all measurements.

To evaluate IB stability, the scattering intensity and hydrodynamic diameter were monitored over time. Data analysis followed the methodology described by Castellanos-Mendoza et al. [49].

### 4.9. Field Emission Scanning Electron Microscopy (FESEM)

Purified AA10 LPMO IBs were diluted 1:20 in ultrapure Milli-Q water and gently vortexed to achieve a uniform suspension. A 1 mL aliquot of each sample was filtered through a 0.22 µm polycarbonate isopore membrane (Merck, Bayswater, Victoria, Australia) mounted on a 13 mm Swinnex filter holder (Merck, Bayswater, Victoria, Australia). Residual particles were removed by rinsing with 500 µL ultrapure water, followed by gentle drying with compressed air.

The membranes were air-dried under laminar flow, mounted on SEM stubs using graphite carbon adhesive tape, and gold-sputter coated with an Emitech K550 coater (Quorum Technologies, Lewes, UK) under an argon atmosphere. Imaging was performed at an accelerating voltage of 5–15 kV using a JSM-7100F field-emission scanning electron microscope (FESEM) (JEOL, Tokyo, Japan). Particle dimensions (length and width) were determined from 20–50 individual IBs per micrograph using ImageJ software (NIH, Bethesda, MD, USA).

### 4.10. Fourier-Transform Infrared (FTIR) Spectroscopy

The secondary structure of IB proteins was analysed by Fourier-transform infrared (FTIR) spectroscopy using a JASCO FT/IR-4700 spectrometer (JASCO, Hachioji, Japan). Aliquots of 5–10 µL of resuspended IBs in Milli-Q water or 1× PBS were deposited onto a zinc selenide (ZnSe) ATR crystal and air-dried for 10–15 min to remove residual moisture. Spectra were collected over 4000–600 cm^−1^ with a resolution of 4 cm^−1^, averaging 32 scans per sample to enhance the signal-to-noise ratio. Background spectra were recorded under identical conditions and subtracted from sample spectra. The crystal was cleaned with 80% ethanol before each run, and reference spectra for Milli-Q water were recorded to remove water interference.

IR spectral deconvolution and amide I band analysis (1700–1600 cm^−1^) were conducted in OriginPro 2025 (OriginLab Corp., Northampton, MA, USA) using Gaussian fitting after baseline correction of the amide I region by applying a straight baseline between 1600 and 1700 cm^−1^. Peak positions were assigned based on established literature ranges and verified using the deconvolution procedure described in the Supplementary Material. Secondary-structure content was determined by normalising the total integrated area of the amide I band to 1 and subsequently summing the areas corresponding to established wavenumber ranges for each structural motif. Bands between 1620–1640 cm^−1^, 1641–1649 cm^−1^, and 1650–1660 cm^−1^ were assigned to β-sheets, random coils, and α-helices, respectively, while β-turns were attributed to 1661–1691 cm^−1^, excluding 1672–1678 cm^−1^ sub-region associated with antiparallel β-sheet contributions [50].

The proportion of each secondary-structure element was calculated as:

Quantity of each element (%) = [*A*_i_/*A*_t_] × 100

where *A*_i_ is the area of each fitted band within the amide I region and *A*_t_ is the total area of all fitted components. Details of the fitting procedure are provided in the Supplementary Material.

## 5. Conclusions

This study shows that robust IB formation is a conserved property of the AA10 catalytic domains examined when expressed in the *E. coli* cytoplasm. Four phylogenetically diverse homologues all partitioned predominantly into the insoluble fraction, forming submicron, β-sheet-rich IBs with ordered nanoscale morphology. Sequence analysis and AGGRESCAN profiling identified conserved aggregation-prone motifs adjacent to the histidine-brace region, suggesting that IB formation may be at least partly encoded within the AA10 catalytic core rather than arising solely from host-related stress. Based on the consistency observed across these phylogenetically diverse AA10 homologues, we propose that strong aggregation propensity may represent a widespread characteristic of AA10 catalytic domains under cytoplasmic expression conditions, although broader sampling will be required to define the full generality of this behaviour across the family. Together with previous work on functional LPMO- and CatIB-based systems, these findings identify AA10 domains as promising natural scaffolds for the rational design of self-assembling biocatalysts and protein-based nanomaterials.

## Figures and Tables

**Table 1:** Relative distribution of soluble and insoluble AA10 LPMO p40 homologs at different IPTG concentrations, determined by densitometric analysis of SDS–PAGE bands shown in Figure 2.

Variant	Fraction ^1^	IPTG (mM)		
		0.1	0.5	1
Kpap_p40_	I	93%	90%	89%
	S	7%	10%	11%
Kari_p40_	I	91%	85%	83%
	S	9%	15%	17%
Alip_p40_	I	95%	92%	88%
	S	5%	8%	12%
Psuf_p40_	I	92%	91%	90%
	S	8%	9%	10%

^1^ Soluble (S) and insoluble (I) protein fractions.

**Table 2:** Retention (%) and pellet quality assessment of AA10 LPMO IBs produced at varying IPTG concentrations.

Variant	IPTG (mM)	Retention (%) ^1^	Pellet Quality
Kpap_p40_	0.1	100	Hard
	0.5	90	Hard
	1.0	62	Moderate
Kari_p40_	0.1	98	Hard
	0.5	89	Hard
	1.0	75	Moderate
Alip_p40_	0.1	55	Moderate
	0.5	25	Soft
	1.0	15	Soft
Psuf_p40_	0.1	93	Hard
	0.5	85	Hard
	1.0	45	Soft

^1^ Retention (%) refers to the proportion of insoluble protein remaining after sequential washing (PBS, 0.1% Triton X-100, and B-PER). Pellet quality descriptors (hard, moderate, soft) reflect visual appearance and ease of resuspension following purification.

**Table 3:** Hydrodynamic diameters and polydispersity indices (PDI) of AA10 LPMO p40 homologue IBs determined by DLS. Values are mean ± SD, n = 3.

Variant	Diameter (nm)	PDI
Kpap_p40_	754 ± 38	0.45 ± 0.04
Kari_p40_	550 ± 50	0.49 ± 0.08
Alip_p40_	863 ± 14	0.54 ± 0.02
Psuf_p40_	559 ± 20	0.51 ± 0.09

**Table 4:** Dimensions of AA10 LPMO IBs derived from FESEM image analysis (mean ± SD, n = 3).

Variant	Length (nm)	Width (nm)
Kpap_p40_	856 ± 19	639 ± 23
Kari_p40_	859 ± 28	602 ± 23
Alip_p40_	789 ± 21	554 ± 19
Psuf_p40_	753 ± 23	582 ± 23

**Table 5:** FTIR-derived secondary-structure composition (mean ± SD, n = 3).

Variant	α-Helix (%)	β-Sheet (%)	β-Turn (%)
Kpap_p40_	41 ± 1.3	50 ± 0.5	9 ± 0.5
Kari_p40_	40 ± 0.9	52 ± 0.3	8 ± 0.5
Alip_p40_	46 ± 1.2	43 ± 0.2	11 ± 0.4
Psuf_p40_	48 ± 1.5	41 ± 0.5	11 ± 0.3

## Data Availability

The data supporting the findings of this study are contained within the article and the Supplementary Materials. Additional data are available from the corresponding author upon reasonable request.

## Supplementary Materials

The following supporting information can be downloaded at: https://www.mdpi.com/article/10.3390/ijms27031188/s1.
