# Supplementary material for: Sequence-Encoded Aggregation of AA10 LPMO Domains as a Basis for Inclusion Body Design
Source: Int J Mol Sci. 2026 Jan 24;27(3):1188. doi: 10.3390/ijms27031188 (PMC12897692; doi:10.3390/ijms27031188)
Supplement: Supplementary file 1 [file ijms-27-01188-s001.zip › ijms-4100147-supplementary.pdf]

Supplementary Material

Sequence-Encoded Aggregation of AA10 LPMO Domains as a Basis for Inclusion Body Design

Ahmad Muaaz Hassan Butt [1] and Anwar Sunna [1, 2,*]

[1] School of Natural Sciences, Macquarie University, Sydney, NSW 2109, Australia; ahmad.butt@hdr.mq.edu.au
[2] Australian Research Council Industrial Transformation Training Centre for Facilitated Advancement of Australia's Bioactives (FAAB), Sydney, NSW 2109, Australia
* Correspondence: anwar.sunna@mq.edu.au

Table S1. Amino acid sequences of the expressed AA10 LPMO p40 homolog constructs. Sequences correspond to the full-length proteins expressed in *E. coli*, including the N-terminal vector-encoded flexible linker. Signal peptides were removed prior to cloning. Asterisks (*) indicate the C-terminal stop codon.

$Kpap_{p40}$<br>MVPSLGGGGSGGGGSGGGGSGSVFPATRTYACYVDGKVNGNGGDLLMTNPACIEAERISGRNQFWNWFGNLISNAGGRHRE<br>IIPDGKLCGPTPTFDGMNQARTDWWTTRVQPGATVTVRYNAWAPHPGTWYLYVTRDGWDPTQPLKWSDLEPVPFNQITNPP<br>INSSGPDGAEYSWQVQLPNKSGRHIIYTIWQRSDSPEAFYNCSDVFFGSGPIEYEFSDPREGG*
$Kari_{p40}$<br>MVPSLGGGGSGGGGSGGGGSGSTFPSTRTHACYVDGKAGGGGDLNPQNPACKAAVAIGGKQPLWDWFGNLISNAGGRHREI<br>IPDGKLCGPTAKYDGYNLARTDWPTTQLQSGAAITFRYNAWAPHPGTWSQYITRDGWNPNQPLKWSDLEATPFNSVTNPPI<br>NGSGPEGAEYTWPGVLPRKTGRHIIYSIWQRSDSPEAFYNCSDVNFGGS*
$Alip_{p40}$<br>MVPSLGGGGSGGGGSGGGGSGSTYPATRTYACYVDGKAGGQGGDLHPTNPACVAAVAEGGKNPLWNWFGNLISNAAGRHRE<br>IIPDGKLCGPTALFDAYNMAHDEWPTTSLQAGSSITIRYNAWAPHPGTWYQYVTKDGWDPSQPLKWSDLEPVPFDTVTNPP<br>INGTGPEGPEYTWTAQLPANKSGRHIIYSIWQRSDSPEAFYNCSDVVFDGG*
$Psuf_{p40}$<br>MVPSLGGGGSGGGGSGGGGSGSVSPATRTYACYVDGRANGGGDLNPTNPACVAAVAQGGKQPLWDFFAVLQSNAGGNHRAI<br>IPDGQLCGGGTTKYAAYNAARTDWPTTQLQSGGTMQFRYNAWAPHPGTWYQYITRDGYDPTQPLKWSDLEATPFDQVTNPP<br>TQGGPSGSEYYWNTRLPVKQGRHIIYSIWQRSDSPEAFYNCVDVQFGG*

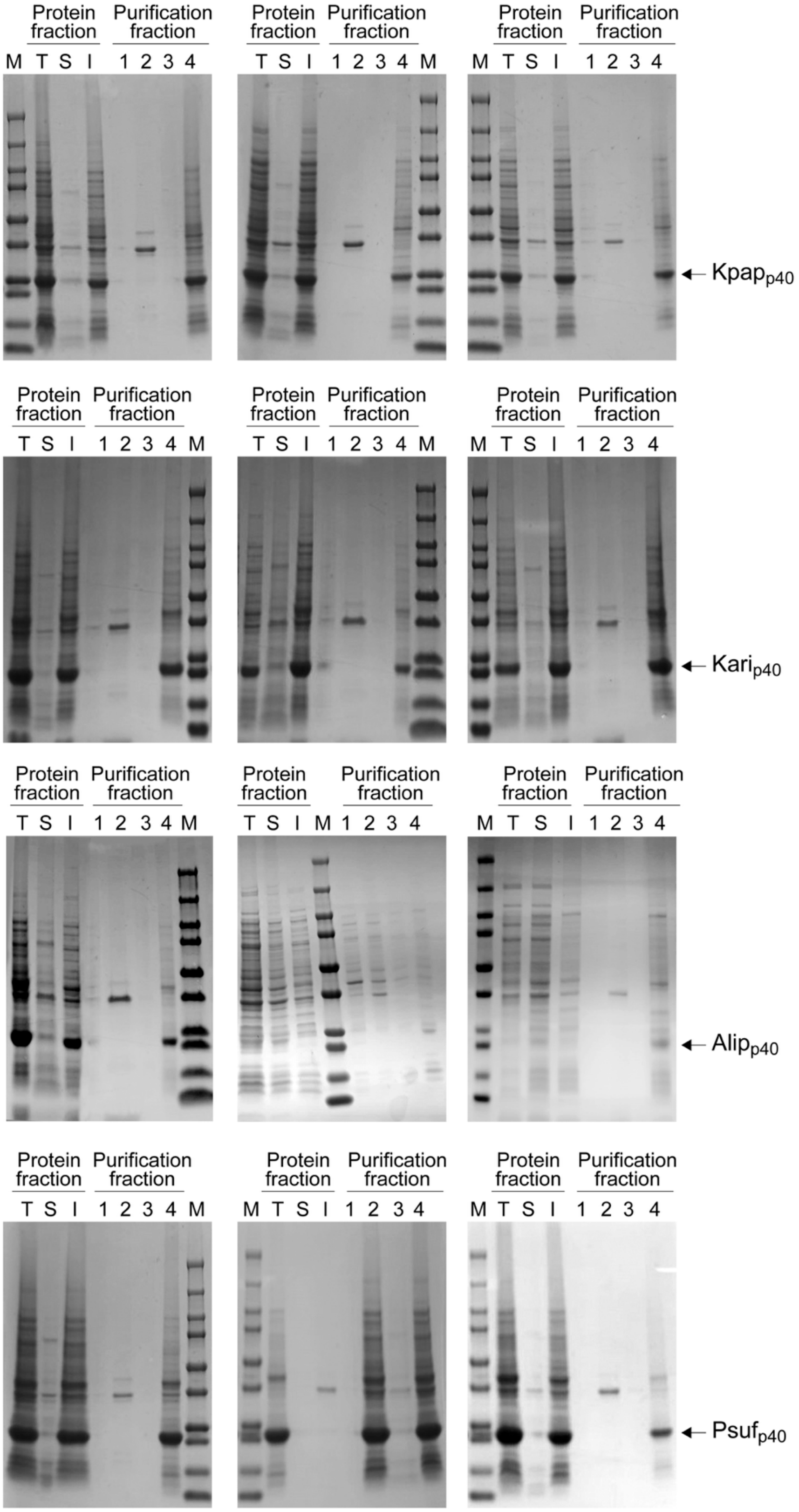

Figure S1. SDS–PAGE analysis of AA10 LPMO (p40 homolog) inclusion bodies (IBs) during sequential washing and purification. Each homolog, Kpap$_{p40}$, Kari$_{p40}$, Alip$_{p40}$, and Psuf$_{p40}$, was expressed *in E. coli* at three IPTG concentrations (0.1, 0.5, and 1.0 mM). Lane assignments are as follows: M, molecular weight marker; T, total protein fraction; S, soluble fraction; I, insoluble fraction; 1, supernatant after PBS wash; 2, supernatant after 0.1 % Triton X-100 wash; 3, supernatant after B-PER treatment; 4, final washed IB pellet. Prominent bands at approximately 22–24 kDa correspond to the expected molecular masses of the recombinant p40 domains.

Table S2. AGGRESCAN-identified aggregation hotspots in AA10 LPMO p40 homologs*.

Variant	Hotspot start	Hotspot end	Length (aa)	Hotspot sequence	a4vAHS (mean)	a4vAHS (max)	HSA (mean)	NHSA (mean)
$Ccel_{p40}$	4	14	11	ATRTYACYVDG	0.153	0.153	1.903	0.173
$Ccel_{p40}$	32	50	19	DALAISGNYQFWNWFGNLI	0.236	0.236	4.858	0.256
$Ccel_{p40}$	94	100	7	ITVRVAW	0.334	0.334	2.481	0.354
$Ccel_{p40}$	105	112	8	GTWYLYVT	0.350	0.350	2.958	0.370
$Ccel_{p40}$	163	170	8	HIIYMIWQ	0.391	0.391	3.285	0.411
$Ccel_{p40}$	180	189	10	NCSDVYFGSG	0.274	0.274	2.936	0.294
$Kpap_{p40}$	4	13	10	ATRTYACYVD	0.164	0.164	1.844	0.184
$Kpap_{p40}$	42	50	9	FWNWFGNLI	0.203	0.203	2.005	0.223
$Kpap_{p40}$	94	99	6	VTVRYN	0.185	0.185	1.231	0.205
$Kpap_{p40}$	106	113	8	GTWYLYVT	0.350	0.350	2.958	0.370
$Kpap_{p40}$	164	170	7	HIIYTIW	0.357	0.357	2.638	0.377
$Kpap_{p40}$	181	190	10	NCSDVFFGSG	0.303	0.303	3.225	0.323
$Kari_{p40}$	31	35	5	AAVAI	0.164	0.164	0.920	0.184
$Kari_{p40}$	41	49	9	LWDWFGNLI	0.188	0.188	1.873	0.208
$Kari_{p40}$	91	99	9	AAITFRYNA	0.189	0.189	1.881	0.209
$Kari_{p40}$	163	169	7	HIIYSIW	0.346	0.346	2.563	0.366
$Alip_{p40}$	5	12	8	TRTYACYV	0.124	0.124	1.155	0.144
$Alip_{p40}$	29	34	6	ACVAAV	0.160	0.160	1.083	0.180
$Alip_{p40}$	42	50	9	LWNWFGNLI	0.238	0.238	2.320	0.258
$Alip_{p40}$	70	76	7	TALFDAY	0.154	0.154	1.220	0.174
$Alip_{p40}$	90	100	11	AGSSITIRYNA	0.147	0.147	1.839	0.167
$Alip_{p40}$	108	113	6	WYQYVT	0.135	0.135	0.928	0.155
$Alip_{p40}$	165	171	7	HIIYSIW	0.342	0.342	2.533	0.362
$Psuf_{p40}$	7	12	6	TYACYV	0.133	0.133	0.918	0.153
$Psuf_{p40}$	28	34	7	ACVAAVA	0.144	0.144	1.146	0.164
$Psuf_{p40}$	41	49	9	LWDFFAVLQ	0.370	0.370	3.510	0.390
$Psuf_{p40}$	108	113	6	WYQYIT	0.139	0.139	0.956	0.159
$Psuf_{p40}$	164	169	6	IIYSIW	0.373	0.373	2.356	0.393
$Psuf_{p40}$	178	188	11	FYNCVDVQFGG	0.138	0.138	1.735	0.158

*Hotspots were identified using AGGRESCAN based on contiguous regions exceeding the aggregation threshold. Reported parameters correspond to block-level aggregation scores calculated by the algorithm.

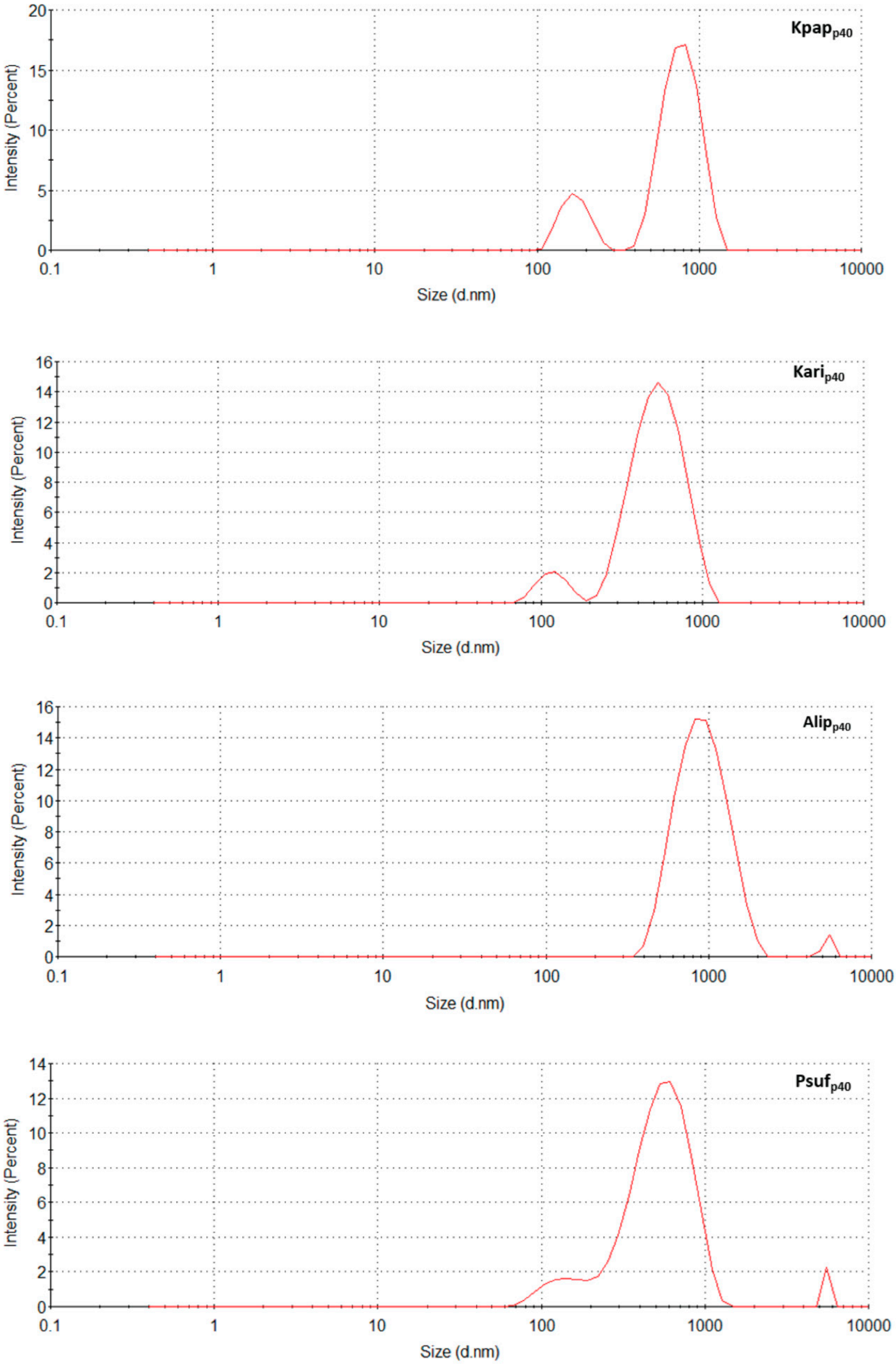

Figure S2. Intensity-weighted dynamic light scattering (DLS) size distributions of AA10 p40 homolog inclusion bodies (IBs). Representative DLS intensity-weighted size distribution profiles of IBs formed by the AA10 p40 homologs Kpap$_{p40}$, Kari$_{p40}$, Alip$_{p40}$, and Psuf$_{p40}$ in aqueous suspension. Mean hydrodynamic diameters and corresponding polydispersity indices (PDI), reported as mean ± SD (n = 3), are summarised in Table 3.

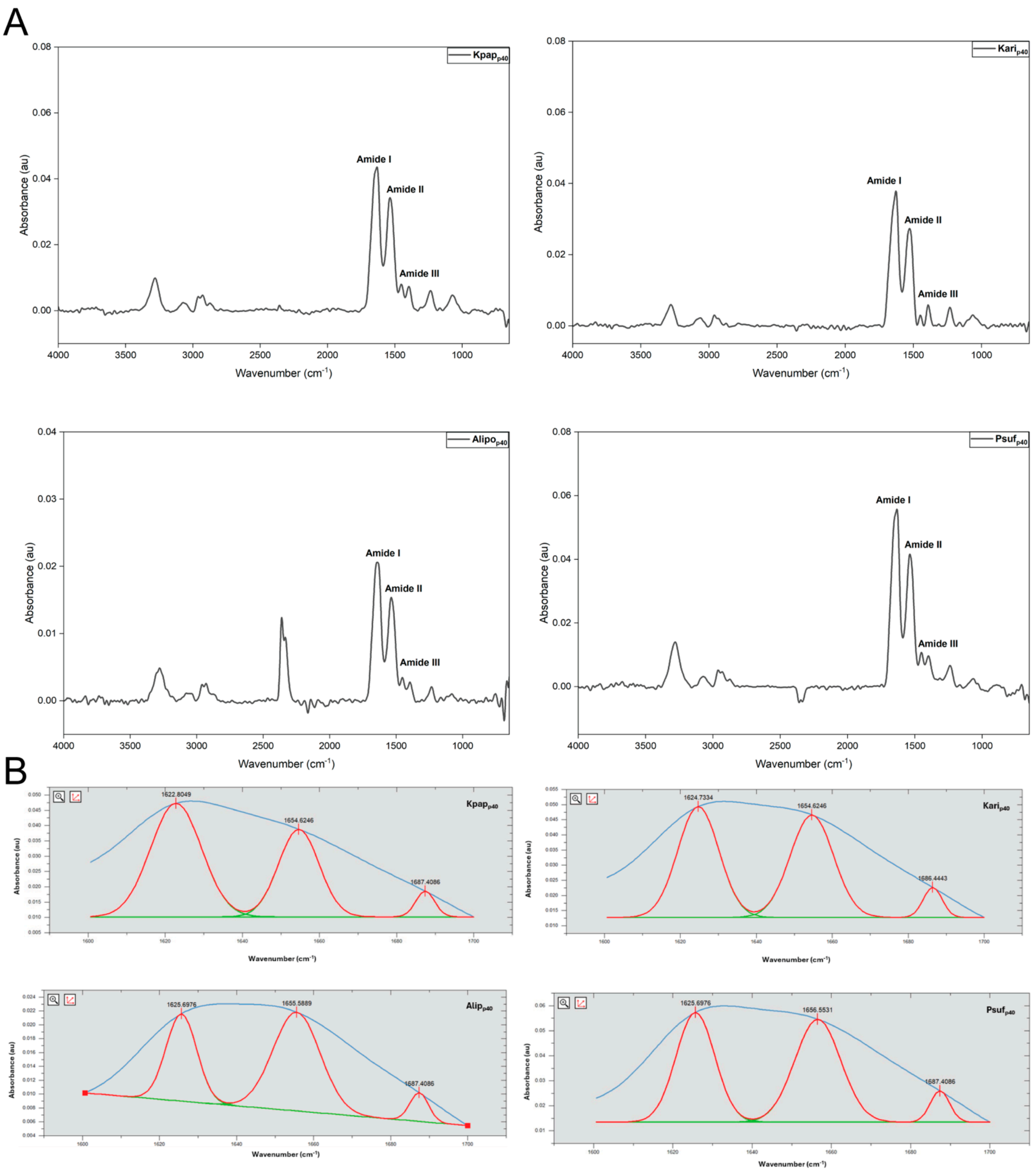

Figure S3. FTIR analysis of secondary-structure features in AA10 p40 homolog inclusion bodies (IBs). (A) Attenuated total reflectance Fourier-transform infrared (ATR-FTIR) spectra of IBs formed by the AA10 p40 homologs Kpap$_{p40}$, Kari$_{p40}$, Alip$_{p40}$, and Psuf$_{p40}$. All spectra display the characteristic amide bands: Amide I (~1650 cm^{-1}), Amide II (~1540 cm^{-1}), and Amide III (~1240 cm^{-1}), reflecting peptide backbone vibrations and the presence of structured protein aggregates. (B) Deconvolution of the Amide I region (1700–1600 cm^{-1}) for each homolog, showing the fitted Gaussian components used to estimate secondary-structure composition. Peaks in the 1620–1640 cm^{-1} region were assigned to β-sheet structures, with additional components corresponding to α-helices, random coils, and β-turns as defined in the Methods.
